# Computational and Experimental Analysis of the Secretome of *Methylococcus capsulatus* (Bath)

**DOI:** 10.1371/journal.pone.0114476

**Published:** 2014-12-05

**Authors:** Stine Indrelid, Geir Mathiesen, Morten Jacobsen, Tor Lea, Charlotte R. Kleiveland

**Affiliations:** 1 Østfold Hospital Trust, Fredrikstad, Norway; 2 Department of Chemistry, Biotechnology and Food Science, Norwegian University of Life Sciences, Aas, Norway; 3 Department of Gastrointestinal and Children Surgery, Institute of Clinical Medicine, University of Oslo, Oslo, Norway; University of Malaya, Malaysia

## Abstract

The Gram-negative methanotroph *Methylococcus capsulatus* (Bath) was recently demonstrated to abrogate inflammation in a murine model of inflammatory bowel disease, suggesting interactions with cells involved in maintaining mucosal homeostasis and emphasizing the importance of understanding the many properties of *M. capsulatus*. Secreted proteins determine how bacteria may interact with their environment, and a comprehensive knowledge of such proteins is therefore vital to understand bacterial physiology and behavior. The aim of this study was to systematically analyze protein secretion in *M. capsulatus* (Bath) by identifying the secretion systems present and the respective secreted substrates. Computational analysis revealed that in addition to previously recognized type II secretion systems and a type VII secretion system, a type Vb (two-partner) secretion system and putative type I secretion systems are present in *M. capsulatus* (Bath). *In silico* analysis suggests that the diverse secretion systems in *M.capsulatus* transport proteins likely to be involved in adhesion, colonization, nutrient acquisition and homeostasis maintenance. Results of the computational analysis was verified and extended by an experimental approach showing that in addition an uncharacterized protein and putative moonlighting proteins are released to the medium during exponential growth of *M. capsulatus* (Bath).

## Introduction

*Methylococcus capsulatus* is a Gram-negative, methane-oxidizing bacterium [1] that has been isolated from marine-, fresh water- and terrestrial habitats. In 2004, the genome sequence of *M. capsulatus* (Bath) strain NCIMB 11132, was published, and results indicated a potential for high metabolic flexibility [2]. Methylotrophs have received considerable industrial interest and a number of patents has been issued for the commercial exploitation of their proteins [3]. *M. capsulatus* (Bath) is the main ingredient in BioProtein (BP), a bacterial single cell protein (SCP) product produced by BioProteins AS (Norway) that serves as a protein source in feedstuff for animals, including salmonids. In 2011, Romarheim et al. showed that dietary inclusion of BP prevents development of soybean meal-induced enteritis in Atlantic salmon (*Salmo salar*) [4]. Recently Kleiveland et al. extended this observation to mammals, when they found a similar effect of BP on dextran sodium sulfate (DSS)-induced colitis in mice [5]. They further demonstrated a comparable effect in mice fed with only *M. capsulatus (Bath)* without the supplementary bacteria present in BP, suggesting that *M. capsulatus* represents the anti-inflammatory principle in BP.

Understanding protein secretion is a key to understanding how bacteria interact with their environment. Secreted proteins are involved in processes such as sensing, signaling, nutrient acquisition and attachment. Following secretion, effector proteins may remain attached to the bacterial surface, may be released to the environment, or may even be injected directly into a host cell. In Gram-positive bacteria, all proteins that are actively translocated across the cell membrane are per definition secreted. In Gram-negative bacteria, in contrast, proteins destined for the surface or the extracellular milieu of the bacterium must first traverse two lipid bilayers, the inner (IM) and outer membrane (OM).

Nine different secretion systems are so far identified in Gram-negative bacteria and have been numerically classified from type I to type IX secretion system (T1SS-T9SS) [6]. These systems range from rather simple systems of few components, to highly specialized multiprotein secretion machineries. Secretion may be achieved in a single step using a contiguous channel spanning both membranes, as is generally regarded to be the case with the T1SS, T3SS, T4SS and T6SS [7]. Alternatively, secretion may be a two-step process in which proteins are first translocated across the IM to the periplasm through general export pathways shared with monoderm bacteria and subsequently translocated across the OM via secretion systems exclusive to Gram-negative species (T2SS, T5SS, T7SS, T8SS, and T9SS).

Proteins contain information aiding the bacterium in assigning them to their correct location. Majority of the proteins secreted by the two-step mechanisms are translocated over the IM via the Sec-pathway in an unfolded state, while the less employed Tat-pathway translocates folded proteins. Whichever pathway being utilized, proteins are generally directed for the IM by an N-terminal signal peptide. In addition to signal peptides, physiochemical characteristics like hydrophobicity, amino acid (aa) charge or polarity are examples of cues to final location. The same type of characteristics can be exploited to predict subcellular location by *in silico* analysis, and a number of prediction programs have been constructed to this end.

Predicting secreted proteins in diderms is more complicated than in monoderm bacteria for two reasons. Firstly, although the presence of a signal peptide is indicative of translocation across the IM, it is not a predictor of final location [8]. Following Sec- or Tat- dependent export, proteins may be anchored to the IM by an uncleaved signal peptide, be released to the periplasm, anchored to the inner face of the OM, integrated in the OM or translocated across the OM by any of the specialized secretion system. Secondly, the numerous mechanisms used by diderms for the translocation of proteins across the OM adds to the complexity, as no universal conserved signal sequence defines secretion across the OM like the N-terminal signal peptide defines IM translocation. *In silico* prediction of protein secretion in Gram-negative bacteria should therefore be tailored to the secretion systems present.

The OM proteome of *M. capsulatus* (Bath) has previously been analyzed using proteomic and computer/bioinformatic approaches [9], [10]. Proteins peripherally associated with the surface was further characterized by Karlsen et al. [11], demonstrating that the surface proteome of *M. capsulatus* (Bath) is highly dynamic. However, less attention has been given to proteins released to the extracellular milieu.

The purpose of this study was to identify the secretome (the secretion/translocation systems and the protein substrates of these transport systems) to extend the knowledge of how *M. capsulatus* (Bath) interacts with its environment. We employed a prediction strategy developed by Romine [12], guided by homology and conserved domains, to predict the secretome of *M. capsulatus* (Bath). In addition to two T2SSs and a T7SS previously identified [13]–[15] we found that putative T1SS, and T5SS are present in *M. capsulatus* (Bath). An *in silico* prediction strategy was used to identify the substrates of each of the *M. capsulatus* (Bath) secretion systems. Analysis of proteins present in the growth medium confirmed that *M. capsulatus* (Bath) secretes adhesion proteins, extracellular enzymes and proteins previously suggested to have functions in copper homeostasis [11]. Furthermore, putative moonlighting proteins and a unique *M. capsulatus* protein not previously recognized as secreted were identified in the growth medium of *M. capsulatus* (Bath).

## Materials and Methods

### Media and growth conditions

*M. capsulatus* (Bath) NCIMB11132 (GenBank accession number AE017282) was cultivated in nitrate mineral salts medium [1] with a head-space of 75% air, 23.75% CH_4_ and 1.25% CO_2_. An overnight pre-culture was pelleted by centrifugation (550 × g at 4°C, 10 min.). The pellet was dissolved in fresh medium to OD_440_ of 0.2 (+/− 0.14) in three biological replicates. Cultures were grown in 350 ml shake flasks in 150 ml medium at 45°C with orbital shaking at 200 rpm.

### Preparation of proteins from culture supernatant

**Figure 1:**
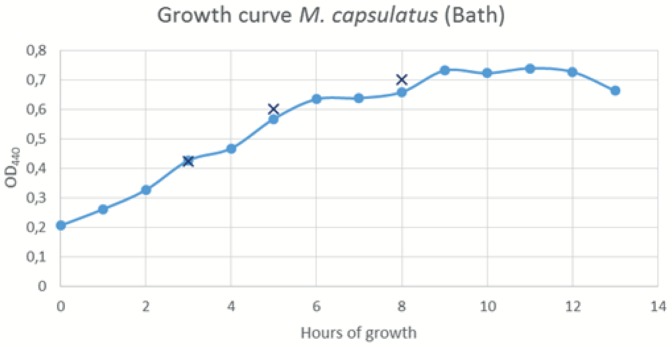
Growth curve showing average OD440 nm for three cultures of *M. capsulatus* (Bath) during growth. Cultures were sampled at OD440 0.424, 0.601 and 0.701 ± 0.15, indicated by X.

Cultures were sampled during early exponential, mid exponential and late exponential growth phase (Figure 1). The bacteria were pelleted by centrifugation (3500 × g at 4°C, 10 min.), the supernatant was sterile filtrated (0.2µm) and PMSF added to a final concentration 0.1 mM. Proteins in the cell free fractions were concentrated by trichloroacetic acid precipitation: Sodium deoxycholate was added to supernatant fractions to a concentration of 0.2 mg ml^−1^, and samples incubated on ice for 30 min. Trichloroacetic acid was added to a final concentration of 16% and samples incubated on ice for 1 hour. Proteins from 10 ml of supernatant were harvested by centrifugation (25000 × g at 4°C, 15 min.), protein pellets washed twice with ice cold acetone and re-centrifuged. Acetone was removed and the pellets air dried.

### In-gel digestion and protein extraction

**Figure 2:**
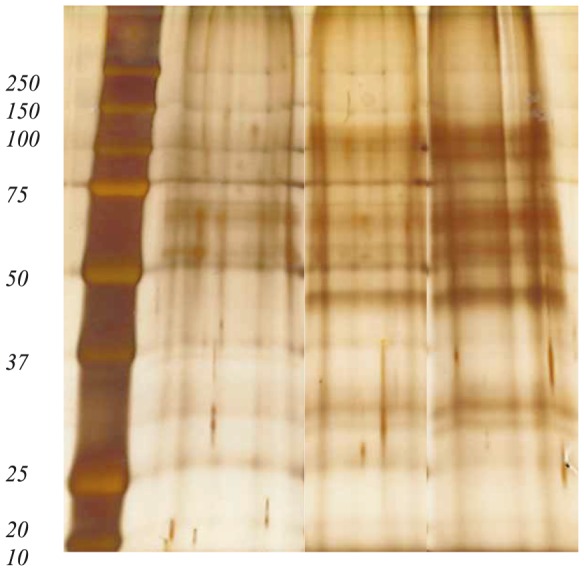
A representative silver stained SDS-PAGE gel showing proteins from cell free culture supernatant of *M. capsulatus* (Bath) culture supernatants from early-, mid, and late exponential growth. Precision Plus Protein Dual Color Standard; molecular weights are indicated in kDa. Proteins precipitated from 10 ml of culture supernatant were applied to the gel.

Proteins precipitated from 10 ml of culture supernatant were resolved in a sample buffer containing 0.125M Tris HCl, 4% SDS, 20% glycerol, 10% 2-mercaptoethanol and 0.002% bromophenolblue (pH 6,8) and separated on a 10% separating gel with a 4% stacking gel [16] (Figure 2). Gels were stained by Coomassie Brilliant Blue R250 and destained. Gel-lanes was either treated as individual pieces or sliced into two pieces, proteins in-gel trypsinated and peptides extracted following the protocol of Shevchenko et al. [17]. Following trypsination and peptide extraction from the gel-pieces, samples were concentrated and desalted using ZipTipC18 (Millipore).

### LC-MS and Database searching

Peptides were analyzed by an ESI-Orbitrap (LTQ OrbitrapXL, Thermo Scientific, Bremen, Germany) mass spectrometer coupled to an Ultimate 3000 nano-LC system (Dionex, Sunnyvale CA) as described in [33]. LC-MS/MS data were analyzed using the Mascot software [18] to search a local database of 2925 *M. capsulatus* protein sequences assuming the digestion enzyme trypsin. Mascot was searched with a fragment ion mass tolerance of 0.60 Da and a parent ion tolerance of 10.0 PPM. Allowed variable modifications specified by Mascot were: S-carbamoyl-methylcysteine cyclization of the N-terminus, deamidation of asparagine and glutamine, oxidation of methionine, acetylation of the N-terminus and acrylamide adducts of cysteine. Protein identifications were validated by Scaffold version 3.3.3, Proteome Software Inc., Portland, Oregon, USA) [19] using the Protein Prophet algorithm [20]. For valid protein identification at least two peptides was required with a probability of ≧95% and a total protein probability of ≧99% and the protein had to be represented in at least two of the three biological replicates.

### Secretome analysis by computational tools

To identify novel secretion systems, the IMG Functional Profile tool [21] was used to search the *M. capsulatus* (Bath) NCIMB 11132 genome using appropriate PFAM and TIGRFAM domains and clusters of orthologous groups. To identify T1SS components the *Escherichia coli* O6:H1 (strain CFT073/ATCC 700928/UPEC) ABC transporter (HlyB) Q8FDZ8 was used as a query in a protein BLAST [27]. The operon structures of the top twenty HlyB hits (E values ≤ 1e-15) were examined using the Prokaryotic Operon DataBase [22]. To predict subcellular location of *M. capsulatus* (Bath) proteins, aa FASTA sequence of 2956 *M. capsulatus* (Bath) proteins were acquired from the NCBI protein database and analyzed using available online prediction programs (table S1) combined in a strategy/pipeline as described in [12]. Lipoproteins were predicted using LipoP 1.0 [23] and LIPO [9]. Protein sequences were analyzed for β-barrel structure using BOMP [24]. Transmembrane helices were predicted by TMHMM server v 2.0 [25] and Phobius [26]. Signal peptide prediction was performed by SignalP4.1 [27] using the high sensitivity option, TatP [28] and TATFIND [29]. PilFind v1.0 [30] was used to predict type IV pilin-like signal peptides. All proteins predicted to contain a signal peptide, that were not predicted to be integral to the IM, and that did not display β-barrel structure or OM domains, were analyzed by Psortb v. 3.0.2, [31] and were searched for homology to secreted proteins by blastp [32] or conserved surface domains by CD-BLAST [33] and Pfam 27.0 [34]. SecretomeP 2.0 [35] was used to predict non-classical protein secretion. Additional evidence, such as functional annotations, genomic context and the localization prediction servers Cello [36] and Psortb v3.0 were consulted to support predictions and resolve inconsistencies.

## Results and Discussion

### The secretion systems of *Methylococcus capsulatus* (Bath)

To account for the substrates of the numerous Gram-negative secretion systems it was important to recognize which secretion systems are present. Several secretion systems are already functionally annotated in *M. capsulatus (Bath)*. T2SS are widespread in Gram-negative species, and a complete classical T2aSS system is present in *M. capsulatus* [13]. Furthermore, a closely related secretion system responsible for type IV pili assembly was previously noted to be present [14]. Following the classification by Chagnot et al. type IV pili systems are categorized as T2cSS [6]. Similarly, a chaperone-usher secretion pathway T7SS responsible for secretion and assembly of fimbrial and non-fimbrial surface structures has previously been recognized in *M. capsulatus* [15].

The IMG Functional Profile tool [21] was used to search the *M. capsulatus* (Bath) genome for novel secretion systems as described in materials and methods. The analysis indicated the presence of a two-partner secretion system (T5bSS), a special case of a T5SS (autotransport) in which the transported protein resides on a different polypeptide chain than the transporter. Interestingly, MCA2226, an OM protein noted by Berven et al. [9] to display sequence similarity to hemolysin activation/secretion protein precursor demonstrates all the characteristics of a two-partner secretion system transporter. MCA2226 is a β-barrel protein that contains a ShlB type POTRA domain and is found in an operon with a typical T5bSS substrate.

Moreover, two putative T1SS were identified in *M. capsulatus* (Bath). T1SS are relatively simple secretion systems that function in a Sec/Tat pathway independent manner. They consist of three components; an OM factor, an IM anchored periplasmic membrane fusion protein and an energy-providing ATP-Binding Cassette (ABC) transporter. In *M. capsulatus* (Bath) MCA1277, an ortholog of the archetypal *Escherichia coli* OM factor, TolC, is present and annotated and is recognized by the domain TIGR01844 (T1SS OM protein, TolC family). However, TolC have other roles besides protein secretion, and its presence in *M. capsulatus* (Bath) is therefore not necessarily indicative of type I secretion [37]. Therefore, to identify additional T1SS components we used the *Escherichia coli* O6:H1 ABC transporter (HlyB) as a query in a protein BLAST [32].

Typically, genes encoding the ABC transporter, membrane fusion protein, and substrates of T1SS are clustered together in the same operon [38]. We therefore examined if any of the top twenty HlyB hits (E values ≤ 1e-15) were found in operon structures characteristic of T1SS. Two of the HlyB hits, MCA1809 and MCA0555, were found in operons next to proteins that contained HlyD domains. Potential T1SS substrates were also found in each operon. Hydrolase function is common to many T1SS substrates [39], and MCA1811 contains an alpha/beta hydrolase domain. The SecretomeP prediction server that predicts non-classical (signal peptide independent) secretion showed a high score (0.94) for this protein giving support to MCA1811 as a secretory protein. MCA0553 display sequence similarity to putative glycosyl hydrolases and contains a discoidin/F5/8 type C domain found in both prokaryotic and eukaryotic proteins involved in various physiological functions such as adhesion, migration and developmental processes [40]. SecretomeP did not predict this protein to be secreted, but a putative secreted protein from *Ignavibacterium album* JCM 16511 was among the closest hits (E value of 0.0) of a protein BLAST [32] using this protein as query. To conclude, our results suggests that together with TolC, MCA1809-MCA1811 and MCA0553-MCA0555 may constitute T1SS components and secreted T1SS substrates in *M. capsulatus* (Bath), but further evidence is needed to confirm the presence of T1SS in *M. capsulatus* (Bath).

T3SS, T4SS and T6SS all form multiprotein nanomachines that inject effectors directly from the bacterial cytoplasm into host cells to promote infection or defense [41]–[43]. Using the IMG functional profile tool we did not find PFAM domains, TIGRFAM domains or clusters of orthologous groups associated specifically with T3SS, T4SS or T6SS, suggesting they are absent in *M. capsulatus* (Bath). Injectisome type secretion systems are typically found in bacteria that live in close interaction with eukaryotes. *M. capsulatus* have, to the best of our knowledge, not been reported to engage in symbiotic relationships, commensal or pathogenic lifestyles. The absence of T3SS, T4SS and T6SS secretion systems in *M. capsulatus* is therefore not surprising.

Similarly, no evidence were found of the T8SS responsible for secretion of curli in enteric bacteria or the T9SS (porphyrin accumulation on the cell surface secretion system) previously suggested to be restricted to the Bacteroidetes phylum [44].

### Genome-wide sub cellular location analysis

In an attempt to identify secreted *M. capsulatus* (Bath) proteins we applied the genome-wide protein localization prediction strategy described by Romine [12]. The strategy resulted in subcellular location prediction for 2956 proteins (table S2). To identify proteins translocated across the IM by Sec- or Tat-translocation pathways all proteins were screened for N-terminal signal peptides using LipoP, SignalP, TatP and PilFind. TMHMM was used to predict integral membrane proteins. All proteins not predicted to be integral to the inner membrane, but that contained a signal peptide, were examined for β-barrel structure or OM domains. If no such domain was found, the protein was searched for surface domains or homology to extracellular proteins. We predicted a periplasmic location for 286 proteins due to the presence of an N-terminal signal peptide and absence of surface domains or detectable extracellular homologs. In total 18 signal peptide-containing proteins displayed surface domains or homology to extracellular proteins and were predicted to be secreted. All proteins predicted to be secreted and the corresponding systems assumed to be responsible for their secretion are indicated in table 1 and are discussed in the following.

**Table 1:** Proteins predicted to be secreted by *M. capsulatus* (Bath).

Secretion system	Gene	Geneproduct	Pfam^a^	Predicted signal sequence
**T1SS**	MCA1811	Hypothetical protein MCA1811	Alpha/beta hydrolase family	No
	MCA0553	Discoidin domain-containing protein	F5/8 type C domain	No
			Amylo-alpha-1,6-glucosidase	
				
**T2aSS**	MCA0875	Serine protease	Peptidase inhibitor I9	SPI
			Subtilase family	
	MCA1028	Endonuclease	DNA/RNA non-specific endonuclease	SPI
	MCA1217	Metalloprotease	Fungalysin/Thermolysin Propeptide Motif	SPI
			Peptidase propeptide and YPEB domain	
			Fungalysin metallopeptidase (M36)	
	MCA2224	PKD domain-containing protein	Receptor for Egg Jelly (REJ) domain	SPI
	MCA2160	Cytochrome c5530 family protein	No^b^/Polycystic Kidney Disease (PKD) domain	SPI
	MCA2328	Hypothetical protein MCA2328	Domain of unknown function (DUF4082)	SPI
	MCA2512	Acid phosphatase	Phosphoesterase family	SPI
	MCA2589	Surface-associated protein	Protein metal binding site	SPI
	MCA2974	PKD domain-containing protein	REJ (Receptor for Egg Jelly) domain	SPI
	MCA0423	Cytochrome c5530	PKD (Polycystic Kidney Disease) domain	SPI
	MCA0338	Cytochrome c5530 family protein	No^b^/Polycystic Kidney Disease (PKD) domain	SPI
	MCA2076	vacJ lipoprotein	VacJ like lipoprotein	SPII
				
**T2cSS**	MCA1510	Fimbrial protein	Type IV pilin N-term methylation site GFxxxE	SPIII
			Pilin	
	MCA0086	Type 4 fimbrial biogenesis protein PilE	Prokaryotic N-terminal methylation motif	SPIII
	MCA0087	Hypothetical protein MCA0087	Neisseria PilC beta-propeller domain	SPI
	MCA0090	Type IV fimbrial biogenesis protein PilV	Prokaryotic N-terminal methylation motif	SPIII
				
**T5bSS**	MCA2227	Hemagglutinin-like protein	Haemagglutination activity domain	SPI
			Filamentous haemagglutinin family outer membrane protein	
				
**T7SS**	MCA0303	Lipoprotein^c^	Spore Coat Protein U domain	SPI
	MCA0306	Spore coat protein, late developmental	Spore Coat Protein U domain	SPI

Proteins predicted to be secreted by *M. capsulatus* (Bath) and the secretion systems responsible are shown. ^a^Significant hits obtained after searches against the Pfam database [34]. ^b^No conserved domain was identified in MCA2160 and MCA0338 searching Pfam 27.0, but Polycystic Kidney Disease (PKD) domains were identified by CD-search [33]. ^c^Although annotated as a lipoprotein, no lipoprotein signal peptide was identified in MCA0303 by the lipoprotein signal peptide prediction tools used in this study.

In general, several of the proteins predicted to be secreted could be found by genome context as their genes are clustered in the genome together with genes encoding components of their secretion systems. Protein transported via T2cSS (type IV piliation system), T5bSS (two-partner secretion system) and T7SS (chaperone-usher secretion pathway) could easily be identified by this approach. Additionally, pilins, the secreted substrates of T2cSS, contain characteristic signal peptides (SPIII), and can be recognized thereby using the PilFind prediction tool [30].

Four *M. capsulatus* (Bath) proteins were predicted as secreted substrates of the T2cSS/Type IV piliation system. Four pilin like proteins contain SPIII signal peptides. The T2cSS/Type IV pili system assemble polymeric fimbrial surface structures, and the major constituent of the structure is referred to as the major pilin [45]. MCA1510 contains a characteristic short leader peptide and is likely the major pilin secreted by the T2cSS. Less abundant pilin-like proteins may also be present in pili and are referred to as minor pilins. Type IV fimbrial biogenesis protein PilE (MCA0086), type IV fimbrial biogenesis protein PilV (MCA0090) and the hypothetical protein MCA0091 FimT homolog are likely minor pilins of the *M. capsulatus* (Bath) T2cSS/Type IV pili system. Reports on the subcellular location of minor pilins, have long been indecisive. Early radiolabeling experiments localized minor pilins to the IM [46], but Giltner et al. [47] demonstrated that the minor pilins PilV and PilE of *Pseudomonas aeruginosa* are incorporated throughout the pilus filament and are thus found on the outside of the bacterial cell. We therefore predict PilV and PilE to be secreted. Additionally the PilY1 homolog (MCA0087), putatively functioning as a tip adhesin of the *M. capsulatus* (Bath) type IV pilus, is expected to be secreted.

Substrates of T5bSS (two-partner secretion systems) are typically large and contain extended signal peptides and a conserved domain that that targets the passenger for translocation across the OM [48]. Interestingly, in *M. capsulatus* (Bath) a large (3349 aa) hemagglutinin-like protein MCA2227 with an extended (43 aa) N-terminal signal peptide, is encoded in an operon with the putative T5bSS transporter MCA2226. In *Bordetella pertussis* filamentous hemagglutinin is a surface-associated adhesin with at least three different binding specificities, including carbohydrate-, heparin sulfate- and integrin binding, and confers adhesion to a variety of cell types [49]. The hemagglutinin-like protein of *M. capsulatus* (Bath) is similarly likely to be involved in cell adhesion.

The two T7SS substrates, putative lipoprotein (MCA0303) and putative sporecoat protein (MCA0306), display sequence similarity to both sporecoat proteins and secreted pilus-adhesins and may form either fimbrial or non-fimbrial surface structures after secretion.

In total we predicted 11 proteins to be substrates of the *M. capsulatus* (Bath) T2aSS (table 1) based on the presence of surface domains or homology to secreted proteins. Enzymes are typical substrates of conventional T2aSS [50], and several of the proteins predicted to be secreted via the *M. capsulatus* (Bath) T2aSS display sequence similarity to recognized secreted enzymes. MCA0875, a serine protease displays sequence similarity to extracellular, thermostable alkaline serine protease of *Thermus* sp. [51] (E value 6e-127 and 58% identity). MCA1028, an endonuclease, displays sequence similarity to an extracellular endonuclease of *Halomonas titanicae* (E value 4e-63 and 42% identity). MCA2512, acid phosphatase displays sequence similarity (E value 5e-167 and 53% identity) to a surface-bound glycoprotein with acid phosphatase activity in *Burkholderia pseudomallei*. [52] and the metalloprotease MCA1217 displays sequence similarity (E value 1e-110 and 37% identity) to a secreted surface metalloprotease of *Psychroflexus torquis* and bacterial and fungal extracellular elastinolytic metalloproteinases of the fungalysin family. Psortb v3.0.2 supported an extracellular localization for these proteins. Moreover, two proteins predicted to be secreted based on the presence of surface domains, display sequence similarity to enzymes: MCA2224 displays sequence similarity to glycoside hydrolases limited to the central region of the protein sequence. MCA2974 displays sequence similarity to fungalysin family proteins limited to the C-terminal half of the protein.

Although the majority of T2aSS substrates are expected to be released to the extracellular milieu after secretion, some are recognized to remain bound to the cell surface, anchored by a lipid moiety, an uncleaved Tat-signal like sequence, or through association with lipopolysaccharides [53]. In *M. capsulatus* (Bath) several of the predicted T2aSS are likely to remain surface bound. The lipoprotein VacJ is surface-exposed in *Shigella flexneri* and has been defined as a virulence factor required for intercellular spread of this pathogen after invasion. VacJ is present and annotated in *M. capsulatus* (Bath) and may be surface associated also in this species. Furthermore, five assumed T2aSS substrates contained the polycystic kidney disease (PKD) domain or the related (REJ) domain found in extracellular regions of metazoan cell-surface proteins, archaeal surface layer proteins [54] and bacterial surface proteins [55]. Indeed, two of the PKD domain proteins was previously identified as peripherally attached surface proteins [11] and was suggested to be implicated in copper homeostasis regulation. Moreover, the copper binding, surface-associated protein MopE (MCA2589) is likely a T2aSS substrate.

### Proteins identified in the growth supernatant

Previous research on the secretome of *M. capsulatus* (Bath) has focused on surface-attached proteins and OM proteins while less attention has been given to proteins released to the extracellular milieu. An exception is the well-studied protein MopE, shown by Fjellbirkeland et al. to occur both as a major *M. capsulatus* OM protein, and as a truncated 336 aa protein secreted into the growth supernatant [56]. Schmid et al. previously characterized the extracellular proteome of the archaeon *Pyrococcus furiosus* by LC-MS/MS-based analysis of culture supernatants [57]. In order to search for proteins released into the growth supernatant, and to confirm the location of predicted secreted *M. capsulatus* (Bath) proteins, we used a similar experimental approach. *M. capsulatus* (Bath) cultures were sampled during exponential growth, proteins were isolated from the culture supernatant and analyzed by LC/MS as described in the materials and methods section.

In an initial attempt, isolating proteins from the stationary phase resulted in identification of a large number of intracellular proteins such as ribosomal proteins, most likely due to cell death and lysis at this growth stage. Consequently, to avoid the problem of protein leakage from dead cells, we collected culture supernatants from *M. capsulatus* (Bath) during exponential growth phase. To gain an overview of whether protein expression and protein release to media changes during growth, culture supernatants were collected from cultures in early-, mid- and late exponential growth phase.

Only 10 proteins were identified in the growth medium from these growth phases (table 2). Seven of the proteins were identified at all sampled time points. Of the 10 proteins found in the growth medium, seven were predicted to contain an N-terminal signal peptides suggesting that they are translocated across the IM.

**Table 2:** Proteins identified in the culture supernatant of *M. capsulatus* (Bath).

Gene	Gene product	MW (kDa)	Early	Mid	Late	Unique exclusive peptides^a^	Total coverage (%) ^b^	SignalP^c^	LipoP/LIPO^d^	TatFind/TatP^e^	PilFind^f^	TMHMM^g^	Phobius^i^	Predicted subcellular location
MCA0338	Cytochrome c5530 family protein	101	Y	Y	Y	59	34	Y	N	N	N	1 TMH^h^	SP	Extracellular
MCA0155	Uncharacterized protein	39	Y	Y	Y	21	35	Y	N	N	N	1 TMH^h^	SP	Periplasm
MCA0875	Serine protease, subtilase family	68	Y	Y	Y	20	15	Y	N	N	N	N	SP	Extracellular
MCA0779	Methanol dehydrogenase protein, large subunit	66	Y	Y	Y	23	25	Y	N	N	N	N	SP	Periplasm
MCA2589	Surface-associated protein	58	Y	Y	Y	15	21	Y	N	N	N	N	SP	Extracellular
MCA1510	Fimbrial protein	14	Y	Y	Y	6	15	N	N	N	Y	1 TMH^h^	1 TMH	Extracellular
MCA1704	60 kDa chaperonin 2	57	Y	Y	Y	14	29	N	N	N	N	N	N	Cytoplasm
MCA1677	Glutamine synthetase	52	N	Y	Y	8	18	N	N	N	N	N	N	Cytoplasm
MCA0707	60 kDa chaperonin 1	57	N	Y	Y	10	34	N	N	N	N	N	N	Cytoplasm
MCA1082	Uncharacterized protein	11	Y	N	N	3	61	Y	N	N	N	1 TMH^h^	SP	Periplasm

Proteins identified from *M. capsulatus* (Bath) culture supernatant from cultures in early, mid and late exponential growth phase. ^a^Column shows the cumulative number of exclusive unique peptides from three biological replicates. Proteins were considered significant if ≧2 peptides were identified with a probability of ≧95% and a total protein probability of ≧99% and the protein was represented in at least two of the three biological replicates. ^b^Total coverage was calculated as the percentage of all aas in a protein, after removing the signal sequence, that is identified from sample spectra. ^c-f^The predicted presence (Y) or absence (N) of: ^c^signal peptide, ^d^lipoprotein signal peptide, ^e^twin-arginine signal peptide or ^f^prepilin-like signal peptide as predicted by SignalP 4.1, LipoP, LIPO, TatFind, TatP and PilFind is indicated. ^g^Column shows the number of transmembrane helixes predicted by the TMHMM prediction program. An asterisk indicates that >10 of the aas found in helixes within the first 60 aas, indicating that TMH may be a signal sequence. ^h^Number of TMH and/or presence of signal peptide predicted by the Phobius prediction program. ^i^Subcellular location predicted *in silico*.

Of the 20 proteins predicted to be secreted by *in silico* analysis, four (20%) were identified in the growth medium of *M. capsulatus* (Bath). In this study we used the same media and temperature conditions as was used by Kleiveland et al. when studying the effects of dietary inclusion of *M. capsulatus* in a murine model of IBD [5]. Under these conditions we did not detect substrates of the putative T1SS, T5SS or T7SS in the *M. capsulatus* (Bath) culture supernatant. Noticeably, proteins that remain surface bound after secretion are missed by our approach. Moreover, both protein expression [11] and membrane retention/release of *M. capsulatus* (Bath) proteins [57] have previously been demonstrated to depend on media and growth conditions. Therefore, the fact that we did not detect substrates of these secretion systems in the culture supernatant does not rule out the possibility that such proteins could be expressed and/or released under different conditions.

As shown in table 2 the presence of the putative T2aSS substrates Surface-associated protein, MopE (MCA2589) and the serine protease MCA0875 was confirmed. The predicted extracellular c-type cytochrome MCA0338 expected to be peripherally attached to the surface of *M. capsulatus* (Bath) [11] was found to be released to the medium by our protocol. Similarly, fimbrial protein MCA1510 predicted to be secreted via a T2cSS was identified at all sampled time points. In contrast, we did not detect other pilin-like proteins, giving support to MCA1510 being the major pilin of *M. capsulatus* (Bath).

There are some disadvantages to *in silico* approaches for prediction of subcellular locations. First, predictions rely on correct annotations and on the existence of domains associated with known protein locations or with homology to proteins of such location. Secondly, such approaches are not suitable for location prediction for proteins that have functions in more than on sub cellular compartment. Of the 10 proteins identified in the culture supernatant of *M. capsulatus* (Bath) six had been assigned a periplasmic or cytoplasmic location by *in silico* analysis.

Three signal peptide-containing proteins identified in the growth supernatant did not contain any surface domains and was predicted to be periplasmic proteins by the *in silico* analysis (MCA0155, MCA0779, MCA1082). Of these MCA1082 was found by CD-blast [57] to contain a periplasmic metal-binding domain. This protein was only detected in culture supernatants during early exponential growth. One possible explanation is that it is not expressed in later growth phases. However, LC-MS/MS is a sensitive method, and the fact that this protein is detected by only three unique peptides from early exponential growth, when few other proteins were present, suggests that it is a periplasmic contaminant that is not masked by the presence of more abundant, true extracellular proteins in these two samples.

Methanol dehydrogenase is generally assumed to be a highly abundant periplasmic protein, but has been found in OM-enriched and surface protein fractions in several studies [9], [11]. The uncharacterized protein MCA0155 was found in one parallel during early exponential growth and in all parallels during mid- and late exponential growth. No homologs were identified for this protein. Since it was consistently identified in the supernatant of all cultures we suggest that MCA0155 is actually an extracellular protein missed by the *in silico* prediction strategy. Further efforts should be made to characterize this protein, as understanding the function of unique extracellular proteins may help in understanding species-specific interactions of *M. capsulatus* (Bath) with its environment. Moreover, three proteins (MCA1677, MCA1704 and MCA0707) without predicted signal peptides were identified from the growth supernatant. All three belong to families of proteins previously described as moonlighting proteins. MCA1677 is a glutamine synthetase. The glutamine synthetase of *Mycobacterium tuberculosis* was among the first bacterial proteins reported to function as a moonlighting protein as it was found to be released into the phagosome in infected human monocytes [58]. In *Lactobacillus crispatus* a signal-less glutamine synthetase is an adhesive moonlighting protein that is surface-attached at acidic pH and released into the buffer at higher pH and that displays binding affinity for collagen, laminin, fibronectin and plasminogen [59]. The two other predicted cytoplasmic proteins identified in the growth supernatant of *M. capsulatus* (Bath) (MCA1704 and MCA0707) are closely related molecular chaperones of the 60 kDa chaperonin (Cpn60) family GroEL. Bacterial Cpn60 has been found to be released or surface associated in a large number of studies and represents the most diverse range of moonlighting activities for any protein family know so far, including functions in adhesion, invasion, biofilm formation and stimulation of host cytokine production as reviewed by Henderson et al. [60]. As all the signal-less proteins found in the culture supernatants belong to protein families previously reported to have functions in both cytoplasm and extracellular locations, and since we did not identify ribosomal proteins or other abundant cytoplasmic proteins in the culture supernatant from exponential growth phase, we suggest that MCA1677, MCA1704, MCA0707 may represent moonlighting proteins secreted by non-classical, signal-less secretion pathways.

### Possible surface associated T2aSS substrates missed by our strategy

As noted, the *in silico* strategy used here relies heavily on correct annotation of transcription start and the existence of well-characterized homologs or conserved domains. Furthermore, our experimental approach will not detect proteins that remain surface attached after secretion. We therefor searched available literature for putative secretion system substrates missed by our approaches.

Karlsen et al. has previously identified 22 proteins in fractions enriched in proteins peripherally associated with the surface of *M. capsulatus* (Bath) [11]. Of these, the authors considered seven to be periplasmic. Of the remaining 15, we found two proteins (MCA0949 and MCA2792) to belong to protein families associated with the periplasm and IM respectively. We further predicted one protein (MCA1738) to be a surface exposed OM β-barrel beyond the scope of this article, and four was predicted to be secreted via a T2aSS (MCA2589, MCA2974, MCA0423, MCA0338). The remaining proteins illustrate well the challenges of *in silico* prediction methods. In our results, these proteins were predicted either to be periplasmic due to lack of extracellular domains/homologs or to be cytoplasmic proteins because no signal peptides were detected by the signal prediction programs used in this study. Indeed, Karlsen et al. noted that three of the proteins predicted to be cytoplasmic proteins by our strategy (MCA0446, MCA0445, MCA0347) are incorrectly annotated and that N-terminal signal peptides are present in the deduced, corrected aa sequences of these proteins [11]. Thus, eight proteins that we have predicted to be located in the periplasm (MCA2590 and MCA2150) or cytoplasm (MCA0765, MCA2259, MCA0347, MCA0421, MCA0445 and MCA0446) may well be T2aSS secreted proteins that go undetected by our methodology.

### Concluding remarks

Here we have combined *in silico* analysis and proteomics to define the secretome of *M. capsulatus* (Bath) to extend the knowledge of how *M. capsulatus* (Bath) may interact with eukaryotic cells, in particular. *In silico* analysis shows that multiple secretion systems are present in *M. capsulatus* (Bath). In addition to previously described T2SS and T7SS, a T5bSS (two-partner) and putative T1SS were identified. The predicted substrates of the diverse *M. capsulatus* (Bath) systems are likely to functions in diverse processes: adhesion, colonization, nutrient acquisition and copper response. Extracellular location of predicted secreted proteins was confirmed by analyzing the growth supernatant of *M. capsulatus* (Bath) for secreted proteins. Furthermore, it was shown that proteins with potential moonlighting function, as well as a protein with no known homologs is released to the medium during exponential growth. Further functional characterization of these proteins should provide new insights in how *M. capsulatus* (Bath) interacts with its environment.

## Supporting Information

Table S1 **Computational tools used to analyze the secretome of** ***M. capsulatus*** **(Bath).**
(XLSX)

Table S2 **Subcellular location predictions for 2956** ***M. capsulatus*** **(Bath) proteins.**
(XLSX)
